# Does status epilepticus in pediatric patients severity score (STEPSS) predict functional outcomes in children admitted to PICU? A retrospective single-center study

**DOI:** 10.1186/s12887-026-06744-3

**Published:** 2026-04-09

**Authors:** Ibtehal Saad Abuelela, Sara Ibrahim Sayed, Marwa Ibrahem Abdelrazic

**Affiliations:** 1 Department of Pediatrics, Faculty of Medicine, Minia University, El-Minia, Egypt; 2 Department of Public Health and Preventive Medicine, Faculty of Medicine, Minia University, El-Minia, Egypt

**Keywords:** Children, Status epilepticus severity score, Pediatric ICU

## Abstract

**Objective:**

To explore the ability of the Pediatric Status Epilepticus Severity Score (STEPSS) for assessment of children in terms of mortality and negative outcomes for those admitted to Pediatric Intensive Care Unit (PICU) with status epilepticus.

**Methods:**

Children admitted to PICU with status epilepticus were included in the study. Descriptive and clinical details, STEPSS, and Pediatric Overall Performance Category Scale (POPC) scores were computed. We examined the performance of STEPSS in predicting adverse outcomes and death.

**Results:**

51 children were involved; most of the children were infants (median = 24 months), and 27.5% had a CNS infection. We found that STEPSS > 2 had poor estimating death rates, with a level of sensitivity of 59%, specificity of 37.9%, a positive predictive ability of 41.9%, a negative predictive ability of 55%, positive likelihood ratio of 0.95, and a negative likelihood ratio of 1.08. The ROC curve (area under the curve: 0.51; 95% confidence interval: 0.36–0.65). STEPSS has a sensitivity of 67.6%, a specificity of 52.9%, a PPV of 74.2%, and an NPV of 45% for poor outcomes (POPC score ≥ 3). The ROC curve (area under the curve: 0.53; 95% confidence interval: 0.38–0.67).

**Conclusion:**

The STEPSS showed poor discriminative ability in our PICU-only cohort, with an AUC close to 0.5 reflecting chance-level performance and non-informative likelihood ratios in the prediction of death and unsatisfactory functioning consequences.

**Supplementary Information:**

The online version contains supplementary material available at 10.1186/s12887-026-06744-3.

## Background

Status epilepticus (SE) is prevalent neurologic emergency, with an imminent fatality rate that varies from 3% to 11% in kids [1]. The disability of SE grows alongside the incidence of seizures that are resistant to the recommended medications [2].

Various therapies highlight the significance of quickly stopping protracted seizures. Refractory convulsive status epilepticus is projected to have a much higher impairment and fatality rate than nonrefractory convulsive status epilepticus, with 42% handicap and 39% fatality rates, particularly after only a short period of monitoring [3].

The longer a seizure continues, the less probable it will subside in the following period [4].

Several techniques have been developed to predict prognosis in SE patients, primarily in adults. The Status Epilepticus Severity Score (STESS) was the first tool to use clinical indicators to forecast outcomes in adult SE patients [5]. This score serves as a measure to estimate patients’ likelihood of survival and risk of mortality [5]. It has been shown to be a reliable predictor of death and the need for intensive therapy [6].

In order to adapt the STESS score to be utilized for children with SE, Sidharth et al. (2019) in Northern India changed one of the STESS score’s variables, specifically the age variable, and assessed it in a sample of children with status epilepticus. It was discovered that the Pediatric Status Epilepticus Severity Score (STEPSS) was helpful in forecasting responses to therapy, death rates, and functioning results for children from the time of initial ER hospitalization to the time of release [7].

On the other hand, patients transferred to an intensive care unit usually experience higher rates of refractory seizures, a mortality rate of 17–67%, and a greater rate of systemic complications. In contrast, for those with status epilepticus (SE), generally [8–10]. It is difficult to treat SE in patients with critical illnesses since, on the one hand, it is frequently obscure and recalcitrant [9, 11].

We hypothesized that STEPSS could predict mortality and functional outcome in children admitted to PICU, as previous studies reported its usefulness when applied to children presented with SE in the emergency department (ER).

## Patients and methods

In this retrospective cross sectional single centered study, we included patients admitted with status epilepticus to the Pediatric Intensive Care Unit (PICU) at the Pediatrics Department, Minia University Hospital. The study center serves as a tertiary referral unit that receives many severe and refractory cases from emergency department, pediatric neurology ward or other hospital PICUs.

The eligibility criteria included the following: (1) Children ranging greater than one month through below eighteen years who were identified with SE, (2) Information obtainable since the first day of PICU enrollment, (3) Children presenting with SE due to primary neurological etiologies, CNS infections, CNS vasculitis, intracranial hemorrhage, metabolic/mitochondrial disorders, and developmental epilepsy. Patients with pre-existing chronic neurological sequelae, including cerebral palsy and chronic neurodegenerative disorders, were also eligible for inclusion.

We excluded from the study children with incomplete or missing: (1) clinical data at admission; (2) information on status epilepticus (SE) management; (3) data required to establish baseline Pediatric Overall Performance Category (POPC); or (4) functional ability follow-up or mortality records. Children with SE secondary to non-neurological systemic conditions—such as trauma, sepsis, shock, hypoxic injury, poisoning, or other multi-organ conditions—were also excluded to minimize confounding factors that could independently influence outcomes in the PICU.

Data collection started from August 2024 to November 2024 for the preceding six months (February 2024 to August 2024). 86 file records had been extracted for patients admitted with SE in that period. 26 records were excluded, as the cause of SE was not due to a primary neurological cause. 60 file records were due to primary neurological causes; however, 9 files were excluded due to missing data, so the study included records for 51 SE patients with primary neurological causes.

This study was approved by the Ethics Committee of Minia University Hospital (approval No. 1250:08:2024) and was performed under the ethical principles of the Declaration of Helsinki and its later amendments. Due to the retrospective nature of the study, the requirement of informed consent to participate was waived by the IRB of Faculty of Medicine, Minia University.

Health care records from PICU medical files for SE children were selected over a comparable study time frame, and the information was retrieved and recorded; only information available since the first day of PICU enrollment was obtained. The patients’ age, sex, epidemiological and medical history, seizing nature, seizing length of time, antiepileptic therapies, and electroencephalogram data were all retroactively evaluated. SE management was carried out in accordance with PICU protocols.

Our practice during management of a child with SE was that all patients diagnosed with status epilepticus (SE) underwent EEG assessment as soon as feasible after stabilization, particularly when there was uncertainty about ongoing seizure activity or in cases of altered consciousness following apparent clinical seizure cessation.

Continuous EEG monitoring was considered for patients with prolonged or refractory SE, or when nonconvulsive seizures are suspected. EEG was also used to guide treatment decisions, including adjustment of antiseizure medication, and is typically maintained for 24–48 h after seizure control, or longer if clinically indicated.

SE has been described as epileptic attacks extending over five minutes and two or more independent epileptic attacks in which awareness wasn’t restored completely [12]. Children who were unresponsive to the initial and second-line medication were classified as having refractory status epilepticus (RSE), whereas episodes that lasted more than twenty-four hours were classified as having super-refractory status epilepticus (SRSE).

The International League Against Epilepsy (ILAE) classifies epileptic attacks as focal, generalized, or undetermined onset. Participants with electrographic abnormalities but no symptomatic convulsions were classified as non-convulsive SE (NCSE), while those who had symptomatic epileptic attacks and EEG epileptic changes were classified as convulsive SE (CSE). CSE was identified when seizure activity ≥ t1 = 5 min; the risk of long-term consequences increases after t2 = 30 min [12].

Participants were assessed for STEPSS using health care files and recorded information; the clinical consequences and death were recorded. Clinical consequences were assessed by comparing the POPC score prior to the SE event to the score when discharging from the hospital. The life expectancy endpoint considered in the current research was through either survival or death while hospitalized.

STEPSS has four variables: state of awareness at admission, age, epileptic attack form, and previous epileptic attacks. It differs from adult STESS by the age variable. STEPSS has been determined by using the following criteria: awareness level (aware or somnolent/confused = 0 degrees, in stupors as well as disturbed consciousness = 1 point), epileptic attack form (seizure type other than generalized equal 0, generalized tonic-clonic seizures (GTCS) equal 1, and SE with no motor events equal 2), past epilepsy background (no history equal 1, positive history equal 0), and the age (if more than 2 years old, given 0, while if lower than two years, given 2) [7]. A STEPSS score > 3 exhibited a high sensitivity (93%) and a high specificity (81%) for undesirable results, according to the first observation [7].

The STEPSS was calculated for each patient based on clinical and laboratory data at the time of PICU admission, according to the original published criteria. One investigator (blinded to outcome data) retrospectively determined the score using information extracted from the medical records. The full STEPSS rubric is provided in Supplementary Table 1.

The Pediatric Overall Performance Category Scale (POPC) score was classified to be normal, and this was scored as 1, minor impairment = 2, moderately impaired = 3, severe impairment = 4, coma = 5, and brain demise = 6. Individuals with POPC scores < 3 will have a good outcome, whereas those with scores ≥ 3 will have poor results [13].

The primary outcomes were hospital mortality and POPC. Hospital mortality is defined as death occurring during the same hospital admission for status epilepticus. All patients received full intensive care and life-supportive management; no cases were transferred for palliative or withdrawal-of-care purposes, as such approaches are not practiced in our institution. The Pediatric Overall Performance Category (POPC) score was used to assess neurological outcomes at hospital discharge. Baseline POPC was reconstructed retrospectively from pre-admission clinical documentation, previous medical records, and, when necessary, information provided by caregivers. Cases with insufficient data to establish baseline POPC were excluded from analyses involving functional outcome comparisons.

### Statistical analysis

Data were analyzed using Statistical Package for the Social Sciences, IBM SPSS 25.0 (IBM; Armonk, New York, USA). Quantitative data were checked for normal distribution by the Kolmogorov-Smirnov test. Median and interquartile ranges were provided for non-normally distributed variables, while qualitative data were expressed by number and percentage. Nonparametric quantitative parameters were analyzed by the Mann-Whitney test. Fisher’s exact test or the chi-square test was used for qualitative data. The probability of less than 0.05 was used as a cutoff point for all significant tests.

MedCalc software version 12.2.1 was used to select the best cutoff point of STEPSS for predicting mortality and POPC by determining sensitivity, specificity, negative predictive value (NPV), positive predictive value (PPV), positive likelihood ratio, and negative likelihood ratio. Receiver operating characteristic curves (ROC) of STEPSS for mortality and unfavorable outcomes were also assessed. Multivariate logistic regression analysis for factors predicting mortality and POPC ≥ 3 was done. The Hosmer-Lemeshow test was calculated to ensure that the model was fit. Also, calibration plots were done for both regression models.

## Results

**Fig. 1:**
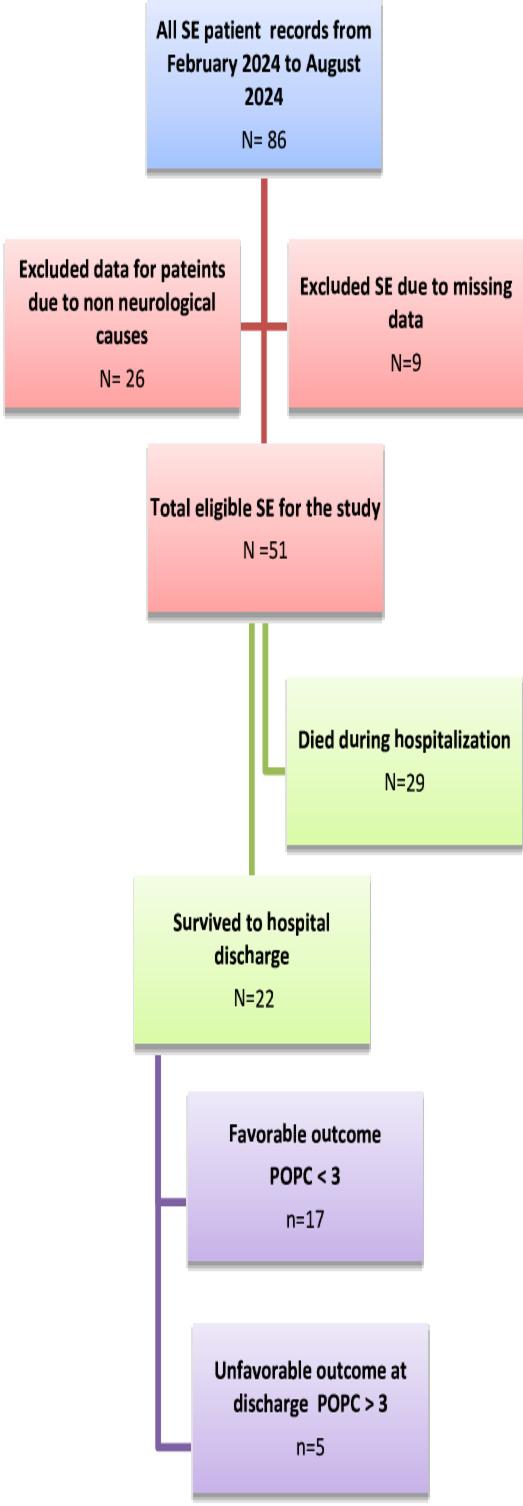
Flow chart for the studied cases

The total number of studied children with SE due to primary neurological causes was 51 children; 17 had POPC < 3, and 32 had an unfavorable outcome (POPC ≥ 3). Of those who had unfavorable outcomes, 5 patients survived with severe disability, and 29 of them died during hospitalization, as shown in Fig. 1.

Thirty-one (60.8%) of the studied cases were aged 2 years or older. The commonest seizure type was GTCS 36 (70.6%), and 3 (5.9%) of patients had nonconvulsive status epilepticus. 31 (60.8%) of patients were without a history of previous convulsions, and 34 (66.7%) patients were in a coma at admission. The median of the STEPSS distribution was 3 [2–4], while 8 (15.7%) of patients had a score of 2, 11 (21.6%) had a score of 3, and 11 (21.6%) of patients had a score of 4. They were admitted to PICU for 10 [7–21] days, and 34 (66.7%) of them had unfavorable outcomes (Table 1).

**Table 1:** Admission data of the studied cases (*n*=51)

Variable	Median (IQ)* or N (%)
Age (years)	
Median (IQ)	2y (9m - 4.5y)
≥ 2 years	31 (60.8%)
< 2 years	20 (39.2%)
Coma	
Alert	17 (33.3%)
Comatose	34 (66.7%)
Seizure type	
Simple-partial, Complex partial, absence, myoclonic	12 (23.5%)
GTCS	36 (70.6%)
NCSE	3 (5.9%)
History of previous seizure	
Yes	20 (39.2%)
No	31 (60.8%)
STEPSS score	
Median (IQ)	3 (2 - 4)
0	3 (5.9%)
1	9 (17.9%)
2	8 (15.7%)
3	11 (21.6%)
4	11 (21.6%)
5	9 (17.9%)
Outcome	
Survived	22 (43.1%)
Died	29 (56.9%)
Treatment response	
Status epilepticus (SE)	3 (5.9%)
RSE	23 (45.1%)
SRSE	25 (49%)
Cause	
Non infectious	37 (72.5%)
Infectious and autoimmune	14 (27.5%)
Length of hospitalization (days)	
Median (IQ)	10 (7 - 21)
Baseline POPC	
Median (IQ)	1 (1 - 3)
Favorable	35 (68.6%)
Unfavorable	16 (31.4%)
Outcome POPC	
Median (IQ)	4 (2 - 6)
Favorable	17 (33.3%)
Unfavorable	34 (66.7%)
Change POPC	
Median (IQ)	1 (0 - 5)
Positive	30 (58.8%)
No change	21 (41.2%)

*Quantitative data are presented by median (Interquartile range), while qualitative data are presented by N (%)

*STEPSS *Pediatric Status Epilepticus Severity Score and *POPC *Pediatric Overall Performance Capacity, *GTCS* Generalized tonic clonic seizures, *NCSE* Nonconvulsive status epilepticus, *RSE* Refractory status epilepticus, *SRSE *Super refractory status epilepticus

Patients were categorized into 2 groups according to previous studies, STEPSS cutoff ≥ 3. On comparing those 2 groups, all patients with STEPSS < 3 had no infectious cause of seizure, while 14 (45.2%) of patients with STEPSS ≥ 3 had an infectious or autoimmune cause of seizure, with a statistically significant difference between the 2 groups. However, we didn’t find any significant difference between those 2 groups regarding mortality or poor functional outcome (Table 2).

**Table 2:** Comparison between patients’ admission data regarding STEPSS cut off value (STEPSS ≥ 3)

Variable	STEPSS < 3N=20	STEPSS ≥ 3N=31	P value
Cause			
Non infectious	20 (100%)	17 (54.8%)	0.001
Infectious and autoimmune	0 (0%)	14 (45.2%)	
Outcome			
Survived	9 (45%)	13 (41.9%)	0.829
Died	11 (55%)	18 (58.1%)	
Length of hospitalization (days)			
Median (IQ)	10 (7 - 21)	10 (7 - 21)	0.712
Treatment response			
SE	2 (10%)	1 (3.2%)	0.181
RSE	6 (30%)	17(54.8%)	
SRSE	12 (60%)	13 (41.9%)	
Outcome POPC			
Favorable	9 (45%)	8 (25.8%)	0.156
Unfavorable	11 (55%)	23 (74.2%)	

Mann- Whitney test was used to compare quantitative data between two groups

Chi-square test was used to compare qualitative data between two groups

(STEPSS): Pediatric Status Epilepticus Severity Score and (POPC): Pediatric Overall Performance Capacity.(SE): status epilepticus . (GTCS): generalized tonic clonic seizures, (NCSE): nonconvulsive status epilepticus, (RSE): refractory status epilepticus, (SRSE): super refractory status epilepticus

*Significant difference (p value ≤ 0.05)

**Fig. 2:**
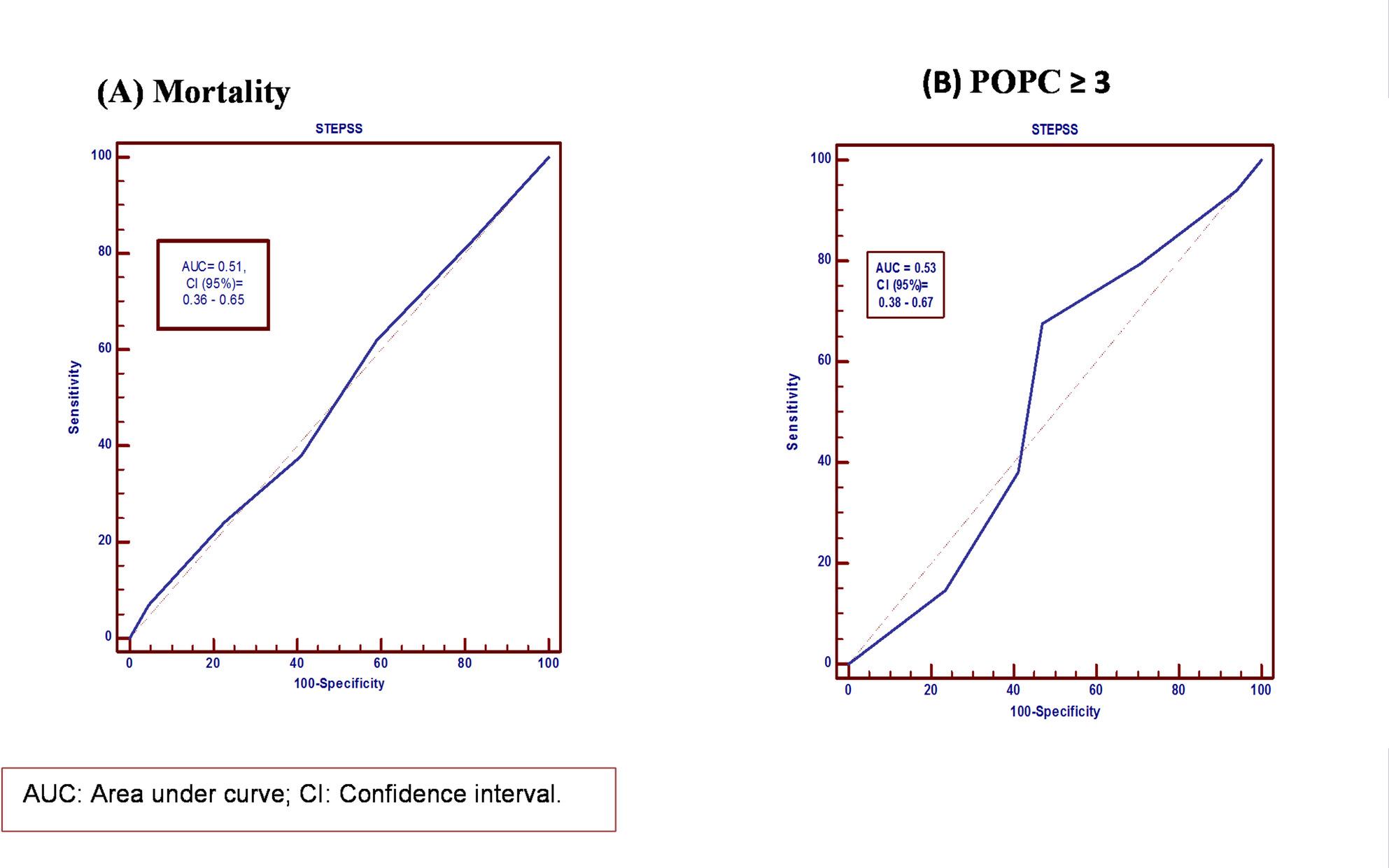
Receiver operating characteristic curve of STEPSS for 30-day mortality (**A**) and unfavorable outcomes **B**

For predicting mortality, our study found that the STEPSS > 2 had 59% sensitivity and 37.9% specificity, which were higher than those for STEPSS > 3. Also, STEPPS > 2 had the highest Youden index (0.031) among the evaluated thresholds. The ROC curve (area under the curve: 0.51; 95% confidence interval: 0.36–0.65) as shown in Table 3; Fig. 2, A. Also, we found that STEPSS predicts negative outcomes (POPC score ≥ 3) at a cut-off of > 2 with higher scores than scores > 3, with sensitivity 67.6%, specificity 52.9%, PPV 74.2%, and NPV 45%, with the highest Youden index (0.20) among the evaluated thresholds, as shown in Table 4.

**Table 3:** Predictive accuracy of STEPSS for mortality

STEPSS	Sensitivity (95% CI)	Specificity (95%CI)	+LR (95%CI)	-LR (95%CI)	PPV (95%CI)	NPV (95%CI)	Yoden index
> 0	95.45% (77.2-99.9)	6.9% (8-22.8)	1.03 (0.90 -1.17)	0.66 (0.06 - 6.81)	43.8% (40.5-47.1)	66.7% (16.2 - 95.4)	0.023
> 1	77.27% (54.6 - 92.9)	24.14% (10.3- 43.5)	1.02 (0.75-1.38)	0.94 (0.34 - 2.57)	43.6% (36.3- 51.2)	58.3% (33.9 - 79.3)	0.014
> 2	59% (36.4-79.3)	37.9% (20.7-57.7)	0.95 (0.61-1.49)	1.08 (0.54 - 2.14)	41.9% (31.5 - 53.1)	55% (38.1- 70.8)	0.031
> 3	40.9% (20.7-63.6)	62.7% (42.3-79.3)	1.08 (0.54 - 2.14)	0.95 (0.61 - 1.49)	45% (29.2 - 61.9)	58.1% (46.9 - 68.5)	0.029
> 4	18.18% (5.2-40.3)	28.7% (64.2-94.2)	1.05 (0.32 - 3.47)	0.99 (0.76 - 1.28)	44.4% (19.5 - 72.5)	57.1% (50.7- 63.3)	0.009
> 5	0 (0-15.4)	100% (88.1 – 100)	-	1	-	56.9% (56.9- 56.9)	

*+*LR* Positive likelihood ratio, –*LR* Negative likelihood ratio, *PPV* Positive predictive value, *NPV* Negative predictive value, *STEPSS* Pediatric Status Epilepticus Severity Score

**Table 4:** STEPSS's predictive performance for Outcome POPC ≥ 3

STEPSS	Sensitivity (95%CI)	Specificity(95%CI)	+LR (95%CI)	-LR (95%CI)	PPV (95%CI)	NPV (95%CI)	Yoden index
> 0	94% (80.3-99.3)	5.8% (0.1- 28.7)	1.00 (0.86 - 1.16)	1.00 (0.097 -10.27)	66.7% (63.4 -69.8)	33.3% (4.6 - 83.7)	
> 1	79% (62.1 - 91.3)	29.4% (10.3 - 56)	1.12 (0.79 - 1.6)	0.70 (0.26 - 1.88)	69.2% (61.3 - 76.2)	41.7% (21 - 65.8)	0.088
> 2	67.6% (49.5 -82.6)	52.9% (27.8-77)	1.44 (0.83 - 2.5)	0.61 (0.32 - 1.18)	74.2% (62.3 -83.4)	45% (29.7 - 61.3)	0.20
> 3	38.2% (22.2 - 56.4)	58.8% (32.9 - 81.6)	0.93 (0.46 - 1.89)	1.05 (0.65 - 1.69)	65% (47.7 - 79.1)	32.3% (22.8 - 43.4)	0.02
> 4	14.7% (5 - 31.1)	76.5% (50.1 - 93.2)	0.62 (0.19 -2.03)	1.12 (0.83 - 1.5)	55.6% (27.8 - 80.3)	31% (25 - 37.7)	0.088
> 5	0 (0 -10.3)	100% (80.5-100)	-	1.00 (1-1)	-	33.3% (33.3 - 33.3)	

*+*LR* Positive likelihood ratio, –*LR* Negative likelihood ratio, *PPV* Positive predictive value, *NPV* Negative predictive value, and *POPC* Pediatric Overall Performance Category Scale

The area under the curve was 0.53 [95% CI: 0.38–0.67] (Fig. 2, B). Both the > 2 and > 3 cutoffs yielded uninformative likelihood ratios, indicating that the score could not reliably distinguish between survivors and non-survivors.

Multivariate logistic regression analysis for factors predicting mortality was done to find that STEPSS > 2 had the highest odds ratio (CI) of 1.45 (0.38–5.37). For detecting prediction factors for POPC ≥ 3, it was noted that STEPSS > 2 had the highest odds ratio (CI) of 2.53 (0.594–10.83), followed by the presence of coma on admission at 2.33 (0.644–8.45) (Supplementary Table 2).

The p-value was found to be insignificant for the Hosmer-Lemeshow test; it was calculated to be 0.339 for the model of mortality and 0.702 for the POPC ≥ 3 model, indicating that the models adequately fit the data. This was seen after calibration plots were done for both models. (Supplementary Figs. 1, 2)

## Discussion

In our study, we evaluated the STEPSS score for predicting mortality and functional outcomes in children with status epilepticus admitted to the PICU, classifying patients according to the previously reported cutoff of ≥ 3 from adult [9, 14–17] and pediatric studies [7, 18, 19].

Based on the ≥ 3 cutoff, children with STEPSS ≥ 3 more frequently had infectious etiologies of status epilepticus (45.2%) compared with those with STEPSS < 3, which is consistent with previous pediatric studies [7, 18, 19]. However, no significant differences in mortality or functional outcomes were observed between children with STEPSS ≥ 3 and those with STEPSS < 3. Interestingly, a STEPSS cutoff of > 2 demonstrated greater diagnostic relevance than > 3, which is consistent with findings reported in subsequent pediatric studies [18–20], although cutoff points for STEPSS > 2 for mortality and functional outcome prediction were not significant for outcome prediction.

As the receiver operating characteristic curve for mortality prediction was not performing well (the area under the curve = 0.51, (95% CI: 0.36–0.65), the sensitivity was 59%, and the specificity was 37.9%). In comparison with those estimated by Soydan et al., who reported AUC for mortality = 0.853 and sensitivity ≈ 0.90, specificity ≈ 0.67 at that cutoff [18].

Regarding functional consequences (POPC ≥ 3) although the AUC = 0.53, the Youden index indicated STEPSS > 2 as the optimal cutoff for unfavorable outcome; with a sensitivity of 67.6% a specificity of 52.9%. However, these values were also lower than those of Soydan et al., who found AUC = 0.917 with a sensitivity of 0.90 and a specificity of 0.86 for STEPSS > 2 in predicting poor functional outcomes [18].

The lower performance of STEPSS > 2 in our study may be due to its exclusive use in the PICU, unlike previous pediatric studies [7, 18, 19] that enrolled children at ER admission and followed them through the neurology ward or PICU. Our hospital, the only tertiary-level center in the governorate, received referrals from other PICUs, the ER, and the neurology ward for uncontrolled SE, reflected in 45% of children with RSE, 49% with SRSE, and 66.7% presenting in a coma, compared with 44.3% were stuporous or comatose in the first pediatric STEPSS study [7]. Consequently, STEPSS > 2 was less sensitive for predicting mortality and functional outcomes in PICU-admitted children, likely due to their worse general condition and higher mortality compared with ER-admitted cohorts.

Halawa et al. (2015) reported that lower Modified Glasgow Coma Scale scores at admission (< 8) were a major predictor of mortality, reflecting the severity of convulsive SE and neuronal injury [21]. This aligns with findings by Rossetti et al. [22] and Mayer et al. [23], which showed that children presenting with impaired consciousness had higher mortality, more treatment-resistant SE, longer hospital stays, and lower chances of returning to baseline functional status.

Although our study focused on SE from primary neurological causes, the PICU setting poses challenges that can reduce the performance of scores like STEPSS. Many patients required mechanical ventilation, sedation or anesthesia, vasoactive medications, or experienced hemodynamic or metabolic instability, all of which can confound clinical assessments of consciousness and seizure semiology—key components of STEPSS. Thus, even in neurologically driven SE, critical illness severity and the PICU case mix can impair the accuracy of neurologic scoring tools. Future studies should explore integrating STEPSS with PICU-specific organ dysfunction scores (e.g., PRISM III/IV, PIM3, PELOD-2, or pediatric SOFA) to improve risk stratification in critically ill children with SE.

The low performance of STEPSS in the pediatric intensive care unit in our study is similar to an adult study about SE in a North American ICU that found an AUC of 0.61 for STESS to anticipate hospital mortality [24], and another research encompassing those suffering from refractory SE admitted to ICU reported an AUC of 0.57 [25]. However, adult data should not be extrapolated to children without caution.

Adult studies have attributed the lower effectiveness of STESS in the ICU to the presence of severe systemic illness and hemodynamic instability, which are major contributors to mortality in critically ill patients [26]. Systemic severity scores, such as the Sequential Organ Failure Assessment (SOFA), which evaluates six parameters, have been shown to predict ICU mortality and baseline functional outcomes more accurately than STESS in patients with status epilepticus [26].

In our study, RSE and SRSE were more common, and systemic anesthetic use was higher than in other pediatric studies, contributing to sedation, cardiovascular compromise, and respiratory depression, which increase hemodynamic instability and mortality and are better reflected by systemic severity scores than by STEPSS [27].

Another factor limiting STEPSS performance is the timing of its application: originally designed for use at initial ER presentation, STEPSS is less reliable when applied after PICU admission, following aggressive anticonvulsant therapy, intubation, and sedation, which can obscure key neurological variables such as consciousness and seizure semiology. Our data suggest STEPSS is more informative at the first clinical encounter rather than later in the PICU, where severity is influenced by multiple non-neurological factors.

## Conclusion

In our cohort, the STEPSS did not show meaningful predictive value for outcomes, likely reflecting the highly selected and severe nature of cases admitted to the PICU. Although performance numerically differed between the > 2 and > 3 thresholds, neither cutoff demonstrated clinical utility, with an AUC close to 0.5 reflecting chance-level performance. This suggests that STEPSS, while valuable in less severe SE populations, may not discriminate outcomes effectively in critically ill pediatric patients requiring intensive care and should be combined with other systemic scales that predict mortality in PICU-admitted children with status epilepticus. Further studies and external validation are needed.

### Limitations

This study has several limitations. As a single-center retrospective study, the findings may not be generalizable to other settings or populations. The relatively small sample size may reduce the ability to detect subtle associations. A further limitation of this study is the inclusion of patients with pre-existing chronic neurological sequelae, such as cerebral palsy and chronic neurodegenerative disorders, which may have resulted in higher baseline Pediatric Overall Performance Category (POPC) scores and could have influenced outcome assessments.

Nevertheless, the study provides useful preliminary data on the low ability of STEPSS to predict mortality and outcomes in the pediatric population admitted to PICU with status epilepticus and highlights the need for larger, prospective multicenter studies.

## Supplementary Information

Supplementary Material 1.

## Data Availability

The datasets collected and analyzed are not publicly available but are available from the corresponding author upon reasonable request.

## Abbreviations

SE: Status epilepticus
STEPSS: Status Epilepticus in Pediatric patients Severity Score
POPC: Pediatric Overall Performance Category Scale
PICU: Pediatric intensive care unit
GTCS: Generalized tonic- clonic seizures
CSE: Convulsive status epilepticus
NCSE: Non convulsive status epilepticus
RSE: Refractory status epilepticus
NRSE: Non refractory status epilepticus
