# Supplementary material for: Does status epilepticus in pediatric patients severity score (STEPSS) predict functional outcomes in children admitted to PICU? A retrospective single-center study
Source: BMC Pediatr. 2026 Apr 9;26:324. doi: 10.1186/s12887-026-06744-3 (PMC13085256; doi:10.1186/s12887-026-06744-3)
Supplement: Supplementary file 1 — Supplementary Material 1. [file 12887_2026_6744_MOESM1_ESM.docx]

**Supplementary Table 1:** Status Epilepticus Severity Score (STEPSS) rubric used for scoring in this study.

| Score feature | Status Epilepticus in Pediatric patients Severity Score (STEPSS) | |
| --- | --- | --- |
| Consciousness | Alert or somnolent/confused | 0 |
|  | Stuporous or comatose | 1 |
| Worst seizure type | Simple-partial,Complex partial, absence, myoclonic | 0 |
|  | Generalised-convulsive | 1 |
|  | Non-convulsive,status epilepticus in coma | 2 |
| Age | ≥2 years | 0 |
|  | <2years | 2 |
| History of previous seizures | Yes | 0 |
|  | No or unknown | 1 |
| TOTAL |  | 0-6 |

This table lists all STEPSS variables, definitions, and scoring criteria [7].

**Supplementary Table 2: Multivariate logistic regression analysis for factors predicting mortality and performance for POPC ≥ 3.**

| **Variable** | **Mortality** | | | **Performance for POPC ≥ 3.** | | |
| --- | --- | --- | --- | --- | --- | --- |
|  | **OR** | **95%CI** | **P value** | **OR** | **95%CI** | **P value** |
| STEPSS > 2 | 1.45 | 0.38 - 5.37 | 0.582 | 2.53 | 0.594 - 10.83 | 0.209 |
| Coma at admission | 0.624 | 0.183 - 2.13 | 0.452 | 2.33 | 0.644 - 8.45 | 0.197 |
| Infection etiology | 0.390 | 0.085 - 1.78 | 0.225 | 0.627 | 0.118 - 3.34 | 0.584 |

OR:odds ratio, CI: confidence interval

(STEPSS): Pediatric Status Epilepticus Severity Score and (POPC): Pediatric Overall Performance Capacity.


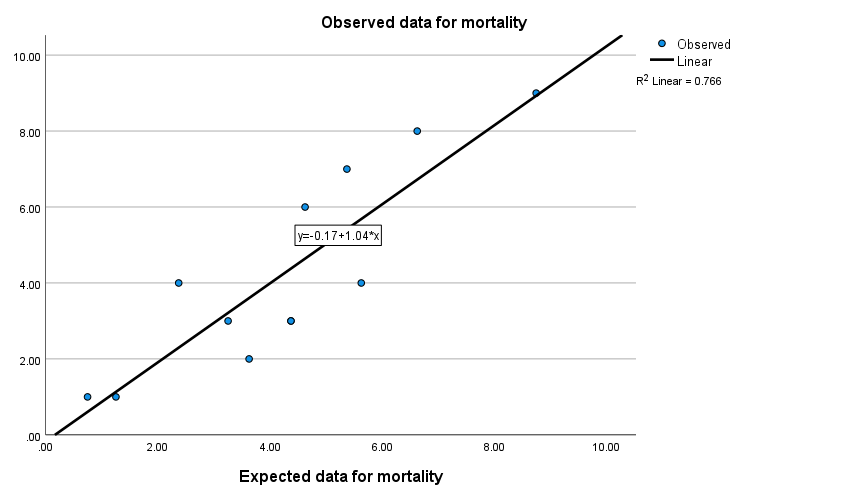


**Supplementary Figure (1): Calibration plot between observed and expected data for multivariate logistic regression for factors predict mortality.**


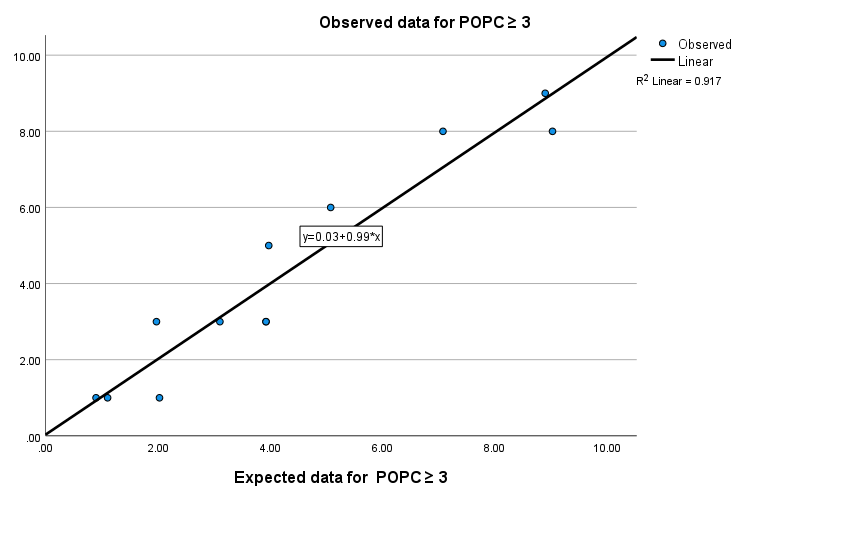


**Supplementary Figure (2): Calibration plot between observed and expected data for multivariate logistic regression for factors predicting performance for POPC ≥ 3.**
